# Transcriptome mining of key genes involved in seasonal changes of total flavonoids in *Eucommia ulmoides* leaves

**DOI:** 10.3389/fpls.2026.1718338

**Published:** 2026-02-17

**Authors:** Siran Wang, Fei Duan, Jun Qing, Yu’e Bai

**Affiliations:** College of Forestry, Inner Mongolia Agricultural University, Hohhot, China

**Keywords:** *Eucommia ulmoides*, flavonoids, leaves, temporal mismatch, total flavonoid content, transcriptome, WGCNA

## Abstract

*Eucommia ulmoides* Oliv. (EU) is a traditional medicinal and edible tree species, and *Eucommia ulmoides* leaves (EUL) are widely exploited for their economic value owing to their high flavonoid content. In EUL, flavonoids contribute to pharmacological activities relevant to human health and to plant functions, but how flavonoid accumulation is coordinated with gene expression during leaf development remains unclear. In this study, we collected leaves at seven developmental stages from April to October, quantified total flavonoid content and profiled the corresponding transcriptomes by RNA sequencing. Transcriptomic analysis identified 14,827 genes, and differentially expressed genes (DEGs) were identified by pairwise comparisons between developmental stages. Their expression profiles were further analyzed using K-means clustering and weighted gene coexpression network analysis (WGCNA) to identify gene sets whose temporal patterns were closely aligned with changes in total flavonoid content. Among the resulting modules, the red module showed a positive correlation with total flavonoid content. Within this module, two key genes, *EU0113959* (CCR1D, cinnamoyl-CoA reductase) and *EU0125338* (PTS1, pterocarpan synthase 1), were most representative, and their expression changes broadly mirrored the seasonal dynamics of total flavonoid content. These two genes were therefore regarded as core candidate genes involved in flavonoid metabolism. Analysis of developmental dynamics revealed that the module eigengene peaked at stage T2 and then gradually decreased, whereas total flavonoid content followed a biphasic pattern, with peaks at T2 and T4 and a modest increase again at later stages. This temporal mismatch between transcriptional activity in the flavonoid-related module and flavonoid accumulation suggests that additional layers of control, such as post-transcriptional regulation, enzyme activity and metabolite transport, are likely to play key roles in shaping flavonoid metabolic profiles in EUL. Overall, this study provides a framework for understanding the developmental regulation of flavonoid biosynthesis in EUL and lays a foundation for optimizing leaf harvest timing and functionally characterizing key regulatory genes.

## 1 Introduction

*Eucommia ulmoides* Oliv. (EU) has been used as a traditional Chinese medicine for more than 2,000 years, with its bark as the main medicinal part and commonly prescribed for the treatment of osteoporosis, threatened miscarriage and age-related disorders. In recent decades, studies have shown that the leaves and bark of EU share highly similar secondary-metabolites profiles. Because the bark has a long growth cycle and debarking can severely damage or even kill trees, the use of *Eucommia ulmoides* leaves (EUL) as an alternative raw material supports more sustainable utilization of *Eucommia* resources and offers broader application potential (Bao et al., 2024).

In line with efforts to promote more comprehensive utilization of EUL, EUL were formally included in the Chinese Pharmacopoeia in 2005 and were subsequently listed as a pilot “medicine and food homology” plant in 2017 (Bao et al., 2024), marking their transition from a traditional medicinal material to a dual-use medicinal and edible resource. EUL are rich in flavonoids, phenolic compounds, lignans and other secondary metabolites (Yang et al., 2024). Among these, flavonoids are of particular interest: on the one hand, they have attracted attention in human health research because of their antioxidant, anti-inflammatory and antitumor activities (Dias et al., 2021); on the other hand, they are among the most common secondary metabolites in plants, contributing to flower color and flavor, enhancing plant tolerance to abiotic and biotic stress and supporting environmental adaptation (Samanta et al., 2011). Thus, flavonoids are regarded as an important biochemical basis for plant adaptation and for the health-promoting functions of plant-derived products (Li and Kang, 2024).

The flavonoids constitute a broad class of natural products with characteristic polyphenolic structures and can be subdivided into major subclasses such as flavones, flavanones, isoflavones and flavonols on the basis of their core skeletons. They are predominantly synthesized via the phenylpropanoid pathway. This pathway starts from phenylalanine, which is deaminated by phenylalanine ammonia-lyase (PAL) to form cinnamic acid, followed by 4-hydroxylation by cinnamate 4-hydroxylase (C4H) and subsequent activation to 4-coumaroyl-CoA by 4-coumarate:CoA ligase (4CL). At this point, Chalcone synthase (CHS) then catalyzes the condensation of 4-coumaroyl-CoA with three malonyl-CoA molecules to produce chalcone. After chalcone isomerase (CHI)–mediated isomerization, downstream hydroxylation and redox steps (eg: F3H, FLS, DFR and ANS) channel intermediates into distinct flavonoid subclasses, while glycosylation and acylation further diversify and stabilize the end products (Xu et al., 2025; Chen et al., 2023).

The accumulation and regulation of flavonoids do not rely solely on key enzymes in the pathway. The transcription factors also play a major role, collectively regulating structural-gene expression and thereby leading to expression patterns that vary across tissues and developmental stages. The MBW complex, comprising R2R3-MYB, bHLH and WD40 proteins, acts as a central regulator of the flavonoid and anthocyanin pathways. In addition, transcription factor families such as NAC, bZIP and WRKY have been shown in various species to integrate developmental and environmental signals and thereby modulate the spatiotemporal expression of structural genes in the flavonoid pathway (Li M. et al., 2024; Li et al., 2025).

Flavonoids exhibit highly dynamic accumulation patterns throughout plant growth and development. Studies in Arabidopsis, grapevine, tea plants and various medicinal tree species have revealed substantial variation in flavonoid content and composition across developmental stages and harvest periods: young tissues often accumulate high levels of flavonols and other antioxidant flavonoids to protect rapidly dividing cells from UV radiation and reactive oxygen species, whereas in later developmental stages metabolic flux may shift towards subclasses associated with pigment deposition, defence responses or signal transduction. Seasonal changes and leaf maturation typically alter the relative abundance of flavonols, flavones and anthocyanins, indicating that flavonoid biosynthesis is under stringent stage- and tissue-specific regulation. In spiny jujube (Ziziphus jujuba var. spinosa), integrated metabolomic and transcriptomic analyses across multiple fruit developmental stages revealed sustained, pronounced accumulation of flavonoids and their derivatives, and weighted gene coexpression network analysis (WGCNA) further identified key components of the flavonoid biosynthesis pathway, particularly FLS genes and NAC transcription factor clusters (Li et al., 2024). Together, these studies demonstrate, from both developmental and regulatory perspectives, that flavonoid metabolism exhibits broadly conserved stage-specific control across diverse plant species and can be effectively dissected using multi-omics strategies.

While recent multi-omics studies have begun to elucidate metabolic variation and regulatory mechanisms in EU in different organizational and biological contexts, how flavonoid accumulation is coordinated with transcriptomic programs during leaf development remains unclear. To address this gap, we quantified total flavonoid content across seven developmental stages and integrated these measurements with RNA-seq to identify flavonoid-related gene clusters and co-expression modules.

## 2 Materials and methods

### 2.1 Plant materials and sampling

**Figure 1:**
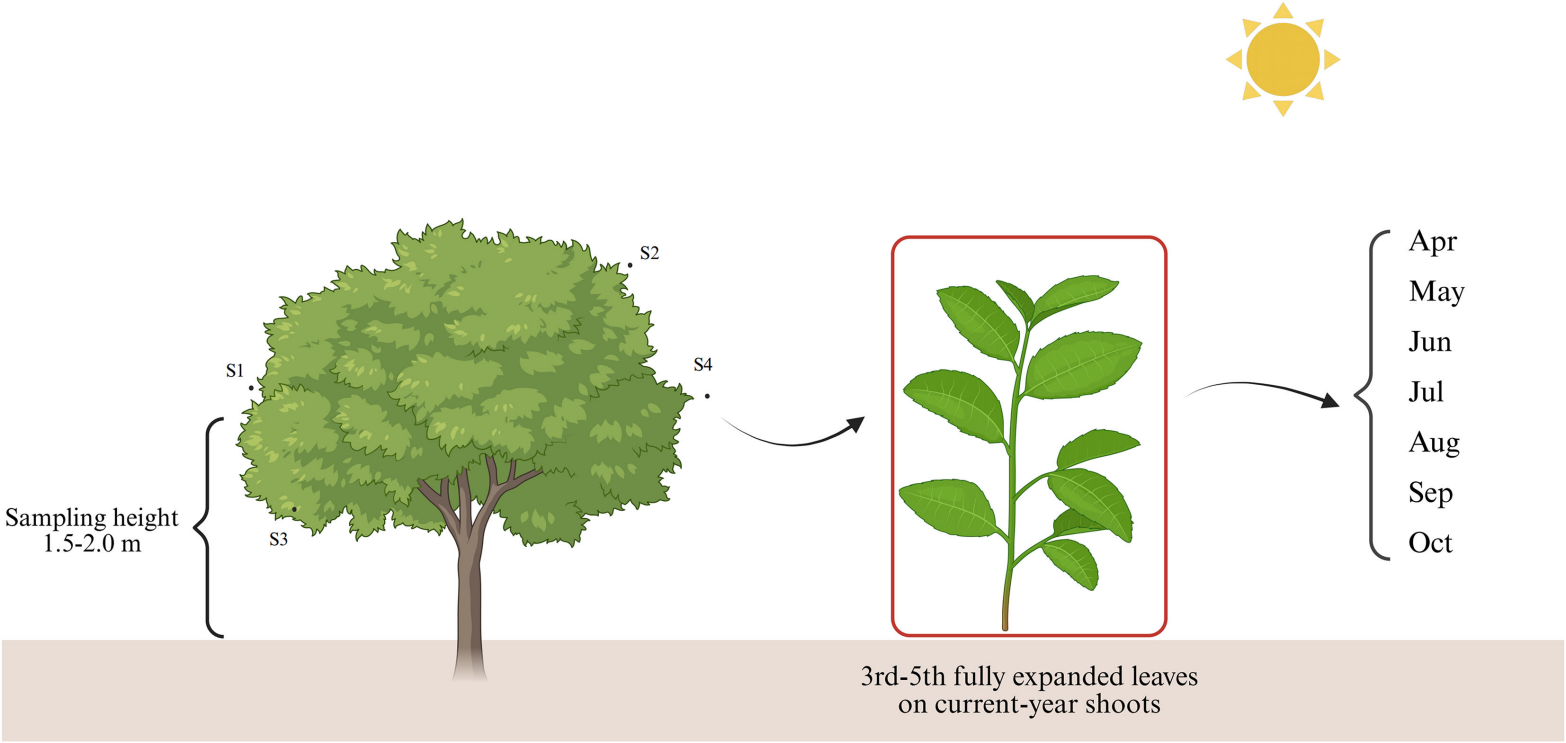
Schematic representation of the sampling strategy for EUL.

We collected leaf samples from 10-year-old EU trees in Baotou, Inner Mongolia. In 2023, sampling was conducted at 28 day intervals from April to October (T1-T7: 27 April, 25 May, 22 June, 20 July, 17 August, 14 September and 12 October). The site has mean annual air temperature of approximately 7-9°C and a mean annual precipitation of about 200–300 mm. Fully expanded, healthy leaves were collected from the outer canopy around the whole crown at a height of 1.5-2.0 m above ground. Within each canopy position, leaves were excised from the 3rd-5th nodes of current-year shoots to minimize variation in developmental stage. For each stage, three independent biological replicates were prepared, each consisting of pooled leaves from three individual trees. After excision, leaf samples were frozen immediately in liquid nitrogen, transported to the laboratory, and preserved at -80°C until determination of total flavonoid content and RNA extraction. The sampling strategy is illustrated in **Figure 1**.

### 2.2 Determination of total flavonoid content

The total flavonoid content (TFC) was measured by a colorimetric method following the Chinese agricultural industry standard NY/T 1295–2007 for the determination of total flavonoids in buckwheat and its products. Fresh leaves (0.10 g) were ground to a fine powder in liquid nitrogen and transferred to a 10 mL centrifuge tube, then extracted with 0.50 mL of 60% (v/v) ethanol. The mixture was mixed thoroughly and incubated in a 60°C water bath for 30 min, and then centrifuged at 10,000 g for 10 min at 25°C. The supernatant was used for the assay.

A standard curve was prepared using rutin as the standard. The regression equation was A = 0.6371C + 0.0353 (R² = 0.991). The amount of flavonoid compounds in each sample was calculated using the following formula:


Total flavonoids (mg / g) = C × V / (M × F)

Where C is the concentration (mg/mL) obtained from the standard curve, V is the extraction volume (mL), M is the sample mass (g), and F is the dilution factor.

### 2.3 RNA-Seq analysis

#### 2.3.1 RNA extraction and sequencing

Total RNA was isolated from EUL with the CTAB–PBIOZOL method, followed by ethanol precipitation and dissolution in 50 μL of DEPC-treated water. RNA concentration was determined on a Qubit Fluorometer (Thermo Fisher Scientific), and integrity was checked with a Qsep400 analyzer (BiOptic).

For library preparation, poly(A)+ RNA was enriched with Oligo(dT) beads, fragmented, and reverse-transcribed using random hexamers. Second-strand synthesis used dUTP in place of dTTP to preserve strand information. The resulting cDNA was subjected to end repair, A-tailing, and adapter ligation, then amplified by PCR. Sequencing was carried out on an Illumina NovaSeq 6000, producing 150 bp paired-end reads. Clean reads were mapped to the chromosome-level *Eucommia ulmoides* reference genome reported by Du et al. (2023).

#### 2.3.2 Functional annotation

The functional annotation of the unigenes assembled from the transcriptome was completed by Metware Company. The annotation database includes NR, SwissProt, Pfam, KOG, GO, and KEGG. Based on the annotation results, the genes were classified by function and used for subsequent GO classification, KEGG pathway enrichment, and functional analysis.

#### 2.3.3 Identification of differentially expressed genes

Differentially expressed genes (DGEs) were screened using DESeq2, with Padj< 0.05 and |log_2_Fold Change| ≥ 1 as the screening thresholds. The resulting DEGs were subsequently subjected to functional annotation and pathway enrichment analysis.

#### 2.3.4 GO and KEGG enrichment analysis

To identify significantly overrepresented biological processes (BP), molecular functions (MF), and cellular components (CC) among the differentially expressed genes, we performed Gene Ontology (GO) enrichment analysis. Kyoto Encyclopedia of Genes and Genomes (KEGG) pathway enrichment analysis was also conducted using KEGG annotations (https://www.genome.jp/kegg/).

#### 2.3.5 K-means clustering analysis

In this study, K-means clustering was used to analyze the expression patterns of flavonoid-related genes in different developmental stages. The expression levels of each sample were used as input, and the final clustering number was set to 6. This classification was used for subsequent data screening and functional analysis.

### 2.4 WGCNA analysis

Weighted Gene Coexpression Network Analysis (WGCNA) was performed on transcriptome data via the Metware Cloud platform (https://cloud.metware.cn). Integrating transcriptome profiles with total flavonoid content allowed us to correlate module eigengenes with the trait and generate expression heatmaps. Modules that were significantly associated with EUL development were identified, and key genes were determined through network visualization.

Then, the module’s module eigengene (ME) is calculated for the modules of interest, which is the first principal component (PC1) of the gene expression matrix for each module. The ME values are then examined for Pearson’s correlation with the total flavonoid content of the corresponding samples, i.e., the first principal component (PC1) of the gene expression matrix for each module. To verify the robustness of the results, leave-one-out cross-validation (LOOCV) is introduced, i.e., the ME is recalculated by removing one sample at a time to assess the stability of the module-trait association.

### 2.5 qRT-PCR analysis

The RNA extracted from each sample was reverse transcribed into cDNA using HiScript III All-in-One RT SuperMix Perfect (Vazyme, Nanjing, China). Taq Pro Universal SYBR qPCR Master Mix (Vazyme, Nanjing, China) was used for qRT-PCR with an ABI Q1 system. Primer sequences are listed in **Table 1**. GAPDH was used as the internal control, and the 2−ΔΔCt method was used to analyze the gene expression data.

## 3 Results and discussion

### 3.1 Seasonal variation of total flavonoid content

**Figure 2:**
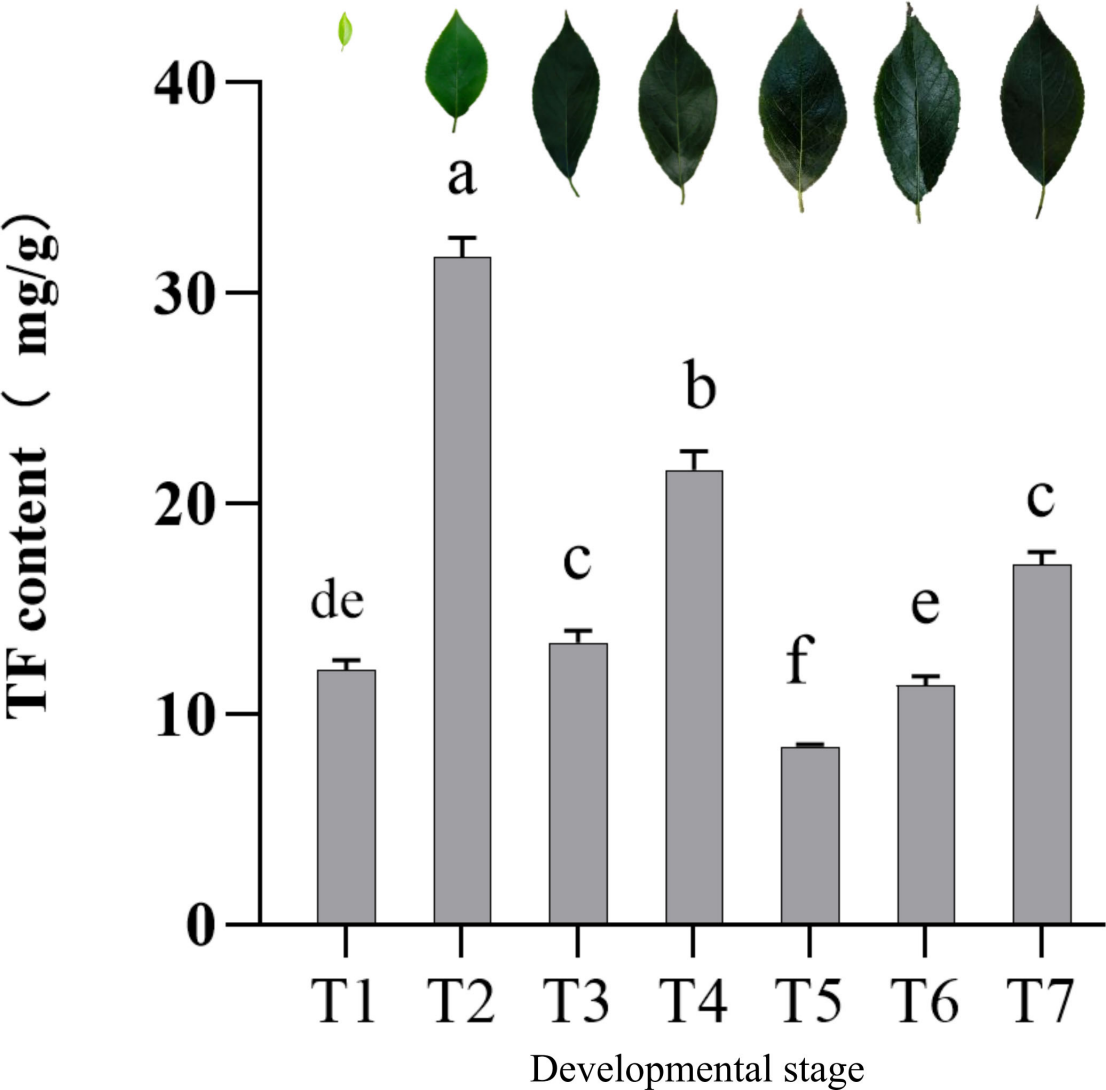
Total flavonoid content in EUL at different developmental stages.

Flavonoids are the main bioactive compounds in EUL, and their levels change during growth and development. Accordingly, we systematically quantified total flavonoid content in EUL across seven developmental stages. As shown in **Figure 2**, total flavonoid content varied across developmental stages. It increased rapidly from T1 to T2, reaching a maximum at T2. And then decreased sharply at T3. A second, smaller peak occurred at T4, followed by a decline to a minimum at T5. Subsequently, total flavonoid content rose slightly at T6 and T7, forming a modest pre-winter peak.

### 3.2 Transcriptomic analysis of EUL at different developmental stages

#### 3.2.1 RNA-seq data quality

**Figure 3:**
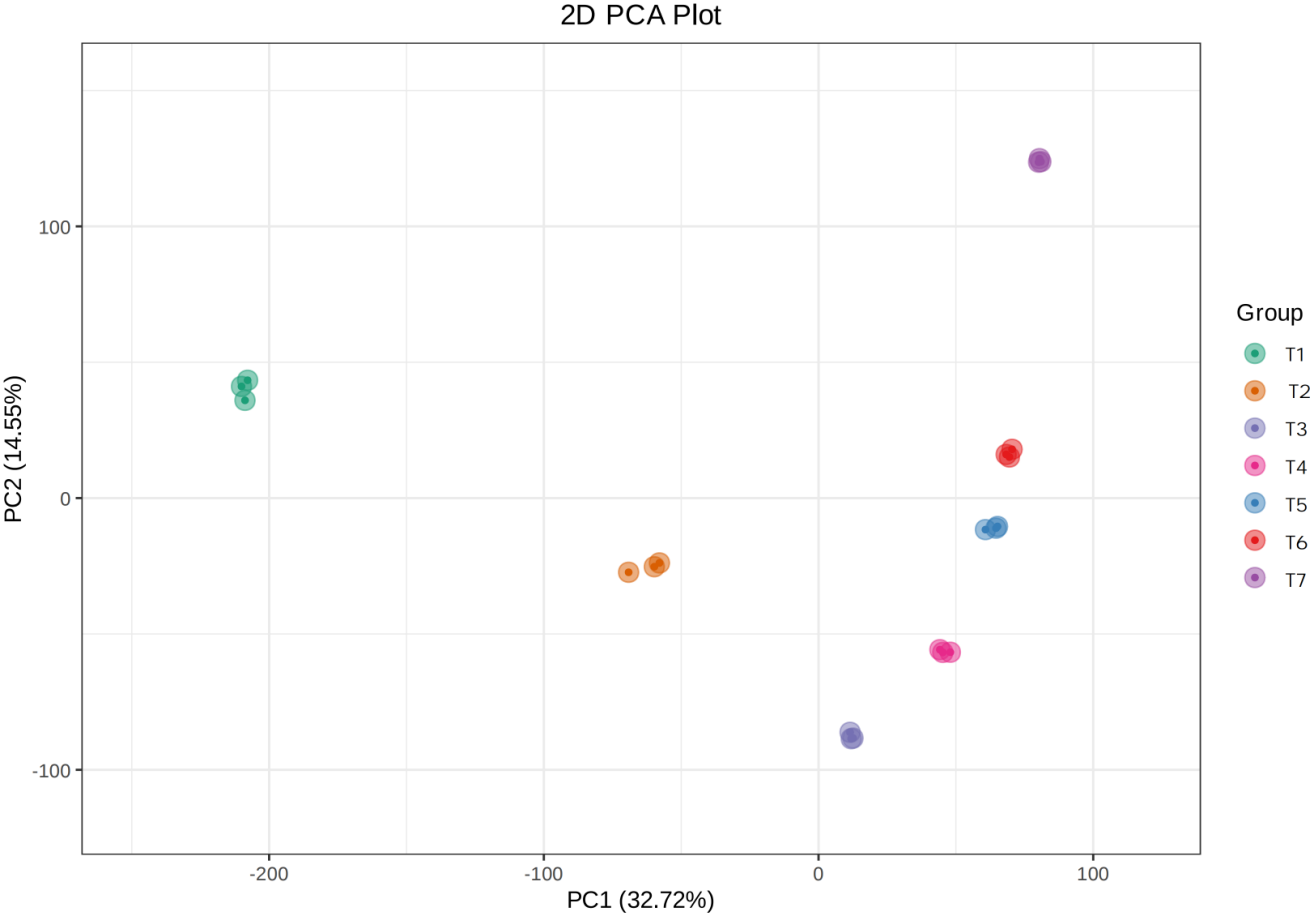
Total flavonoid content in EUL at different developmental stages.

The total flavonoid content in EUL changes markedly across developmental stages. We therefore performed RNA-seq on leaves collected at seven developmental stages T1-T7 to characterize the transcriptional patterns underlying these changes and to identify candidate regulatory genes involved in flavonoid metabolism. In terms of sequencing quality, sequencing generated a total of 134.79 Gb of clean data. The GC content was approximately 47%, and more than 94% of bases had quality scores of Q30 or higher (**Table 2**). After quality control, more than 92% of clean reads mapped to the reference genome, thereby supporting the accuracy and completeness of the assembly. Within each developmental stage, Pearson correlation coefficients between biological replicates were high, and principal component analysis (PCA) showed that the three biological replicates for each stage clustered tightly, confirming the robustness and reproducibility of the dataset. PC1 and PC2 explained 32.72% and 14.55% of the total variance, respectively, and samples from different developmental stages were clearly separated in the PCA plot (**Figure 3**), indicating strong stage-specific differences in gene expression.

#### 3.2.2 Identification of DEGs across developmental stages of EUL

**Figure 4:**
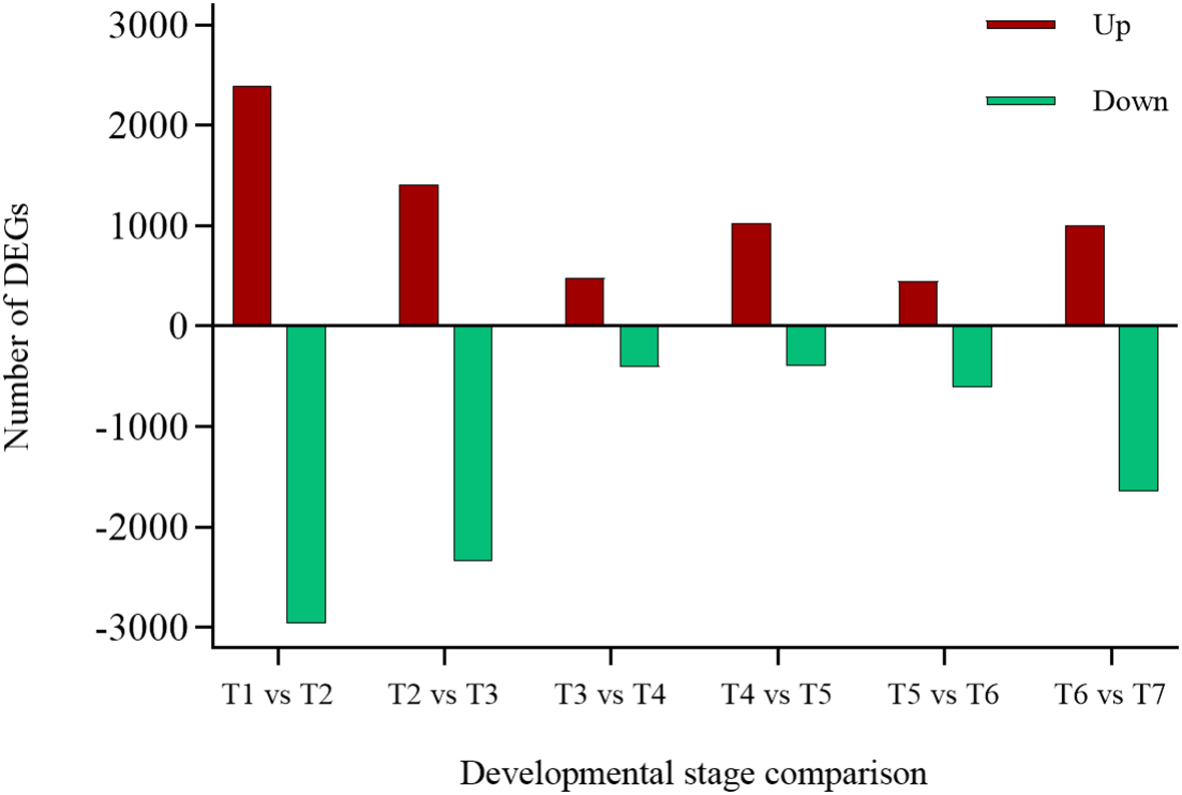
DEGs across consecutive developmental stages of EUL.

RNA-seq analysis of EUL at seven developmental stages detected 14,827 genes with reliable expression across all samples. In pairwise comparisons of the six neighboring stage pairs (T1-T2, T2-T3, T3-T4, T4-T5, T5-T6 and T6-T7), we identified 9,370 DEGs by DESeq2 using with Padj< 0.05 and |log2FC| ≥ 1 (**Figure 4**; **Supplementary Table S1**). The largest transcriptional shift occurred in the T1 vs T2 comparison, with 2,391 up-regulated and 2,964 down-regulated DEGs (5,355 in total), whereas the smallest difference was observed between T3 and T4, with 485 up-regulated and 406 down-regulated DEGs (891 in total). The other stage comparisons followed a similar pattern. The T2 vs T3 transition, which also corresponds to a rapid growth phase, showed a high number of DEGs, whereas T4 vs T5 and T5 vs T6 involved relatively few transcriptional changes, indicating a more stable period. DEG numbers then increased again in T6 vs T7, so that early (T1-T3) and late (T6-T7) stages showed higher DEG abundance than the mid-developmental stages (T3-T6), indicating more pronounced transcriptional reprogramming at the beginning and end of leaf development. This pattern was consistent with the PCA results, in which samples from early and late stages were more clearly separated than those from intermediate stages.

#### 3.2.3 Pathway enrichment analysis

We next explored the biological functions associated with developmental changes in gene expression by performing GO and KEGG enrichment analysis for DEGs identified in each of the six adjacent stage comparisons. GO enrichment showed that DEGs were mainly associated with broad cellular and metabolic functions, whereas in the MF category binding was the most strongly enriched term across comparisons (**Supplementary Figure S1**).

**Figure 5:**
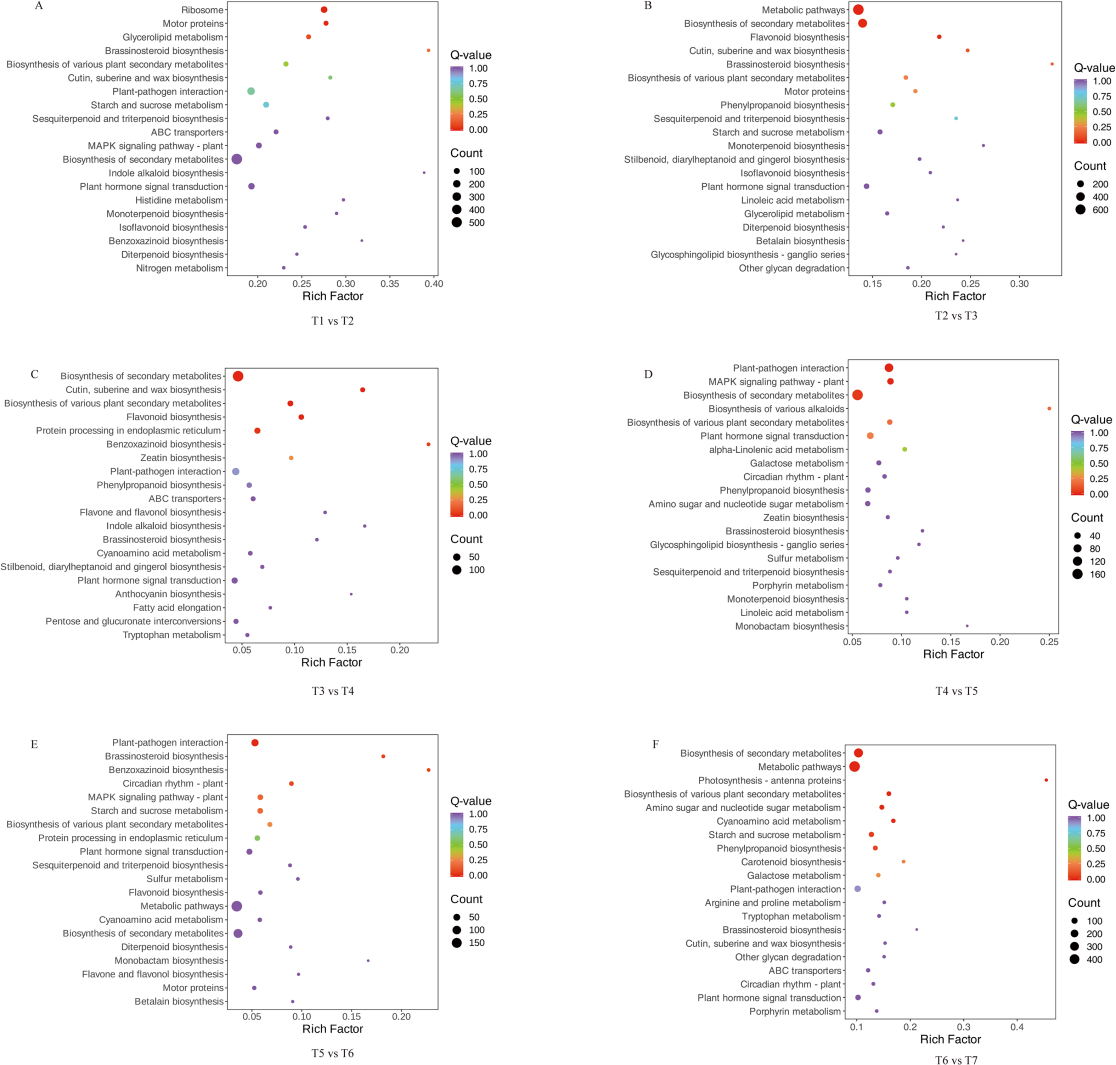
KEGG enrichment for each adjacent stage comparison. Bubble size indicates the number of DEGs; colour indicates the Q-value. Only pathways with Q < 0.05 are considered significant; others are shown for comparison. **(A)** T1 vs T2; **(B)** T2 vs T3; **(C)** T3 vs T4; **(D)** T4 vs T5; **(E)** T5 vs T6; **(F)** T6 vs T7.

In contrast, KEGG enrichment highlighted clear stage-dependent changes in flavonoid-related pathways. In the earliest comparison, T1 vs. T2 only isoflavonoid biosynthesis (ko00943) among flavonoid-related pathways passed the enrichment threshold, and relatively few DEGs mapped to this pathway. In the subsequent comparisons, flavonoid-related pathways showed broader and stronger enrichment. In the T2 vs T3 and T3 vs T4 comparisons, phenylpropanoid biosynthesis (ko00940), flavonoid biosynthesis (ko00941), flavone and flavonol biosynthesis (ko00944) and anthocyanin biosynthesis (ko00942) were all significantly enriched, indicating extensive reprogramming of the core flavonoid backbone together with multiple downstream branches. In the T4 vs T5 comparison, only phenylpropanoid biosynthesis (ko00940) remained enriched among flavonoid-related pathways, whereas in T5 vs T6 flavonoid biosynthesis (ko00941) and flavone and flavonol biosynthesis (ko00944) became enriched again, suggesting renewed adjustment of specialized flavonoid derivatives at later developmental stages. In the T6 vs T7 comparison, phenylpropanoid biosynthesis (ko00940) was again the only enriched flavonoid pathway, while broader pathways such as biosynthesis of secondary metabolites and metabolic pathways showed strong enrichment (**Figure 5**).

#### 3.2.4 Identification of flavonoid key genes

We focused on the 9,370 DEGs to identify genes associated with flavonoid biosynthesis, These DEGs were annotated against the KEGG database, and genes mapped to five pathways that constitute the core network of flavonoid metabolism were selected: phenylpropanoid biosynthesis (ko00940), flavonoid biosynthesis (ko00941), anthocyanin biosynthesis (ko00942), isoflavonoid biosynthesis (ko00943), and flavone and flavonol biosynthesis (ko00944). These flavonoid-related DEGs were then used in subsequent analyses of temporal expression patterns and functional characterization.

##### 3.2.4.1 K-means clustering of flavonoid pathway genes

**Figure 6:**
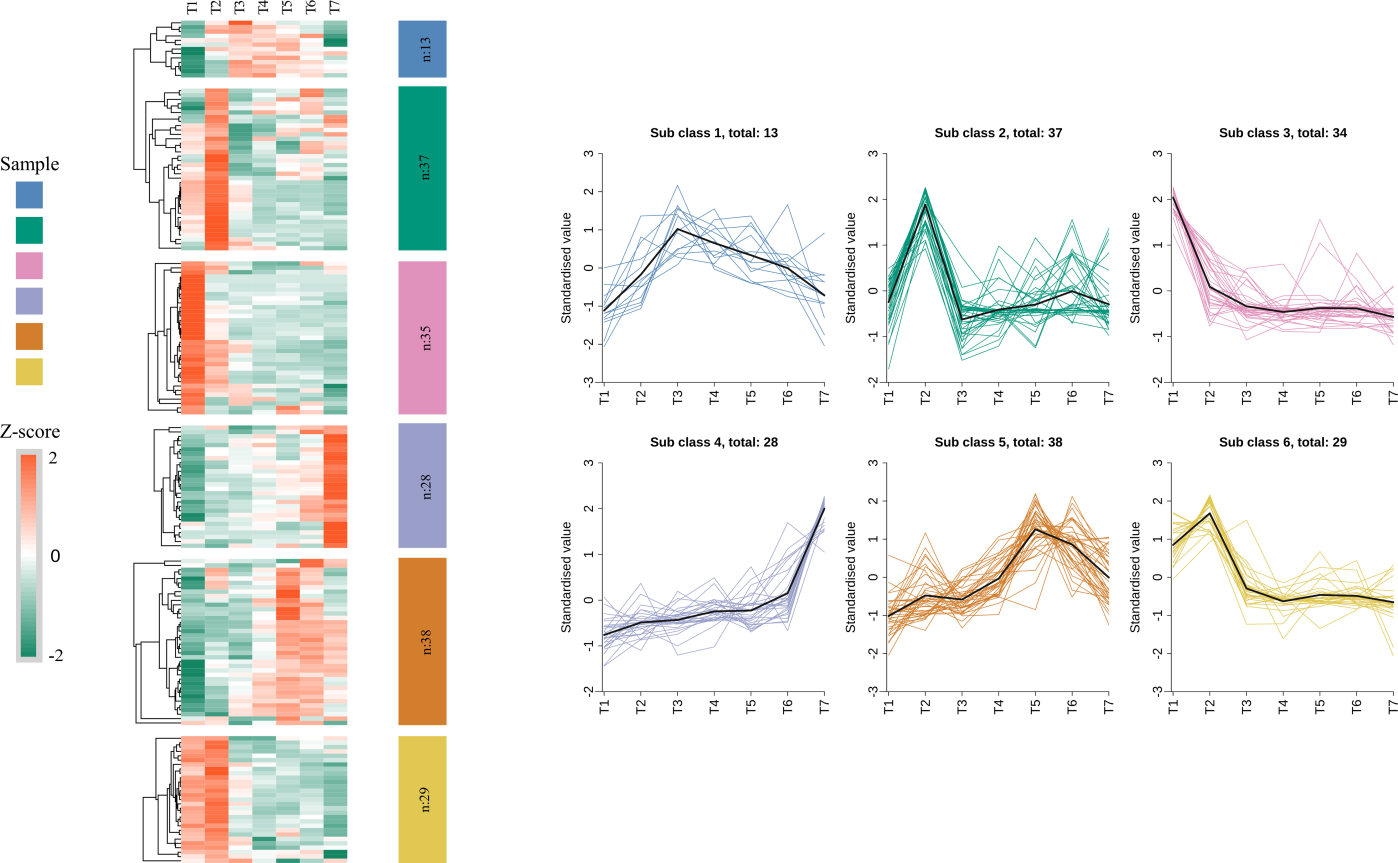
K-means clustering of 179 DEGs related to flavonoid biosynthesis at different developmental stages of EUL. On the left, a heatmap shows the clustering of 180 common differential genes, which were grouped into 6 classes based on K-means clustering. On the right, the expression trends of these genes across five stages of leaf growth (T1-T7) are depicted for each of the 6 classes.

The 180 flavonoid-related differentially expressed genes identified in the previous step were analyzed by K-means clustering based on their temporal expression profiles. These genes were grouped into six subclasses according to their expression patterns across stages T1-T7 (**Figure 6**). For subclasses 1, 2 and 6, gene expression increased and decreased in step with the total flavonoid content. In each of these subclasses expression was highest at T2-T4, which coincides with the main period of total flavonoid accumulation in EUL. These subclasses are therefore likely to be enriched in candidate structural genes and regulatory factors closely associated with flavonoid accumulation in EUL. In addition, genes in subclass 5 showed a gradual increase in expression during leaf development and remained at relatively high levels at the middle and late stages, broadly coinciding with the overall period of flavonoid accumulation.

Notably, subclasses 3 and 4 displayed opposite expression patterns. In subclass 3, transcript levels were highest at the earliest stage (T1) and then declined steadily as the leaves developed. By contrast, subclass 4 showed low expression at early stages, followed by a sustained increase, with maximum levels at the late stages. This contrast suggests that these two subclasses may contain genes with distinct or stage-specific regulatory roles in flavonoid metabolism. We therefore treated subclasses 1, 2, 5 and 6 as prime sources of core genes for flavonoid biosynthesis and regulation, and focused our subsequent functional analyses on genes drawn from these four subclasses.

##### 3.2.4.2 Functional characteristics of flavonoid-related subclasses

**Figure 7:**
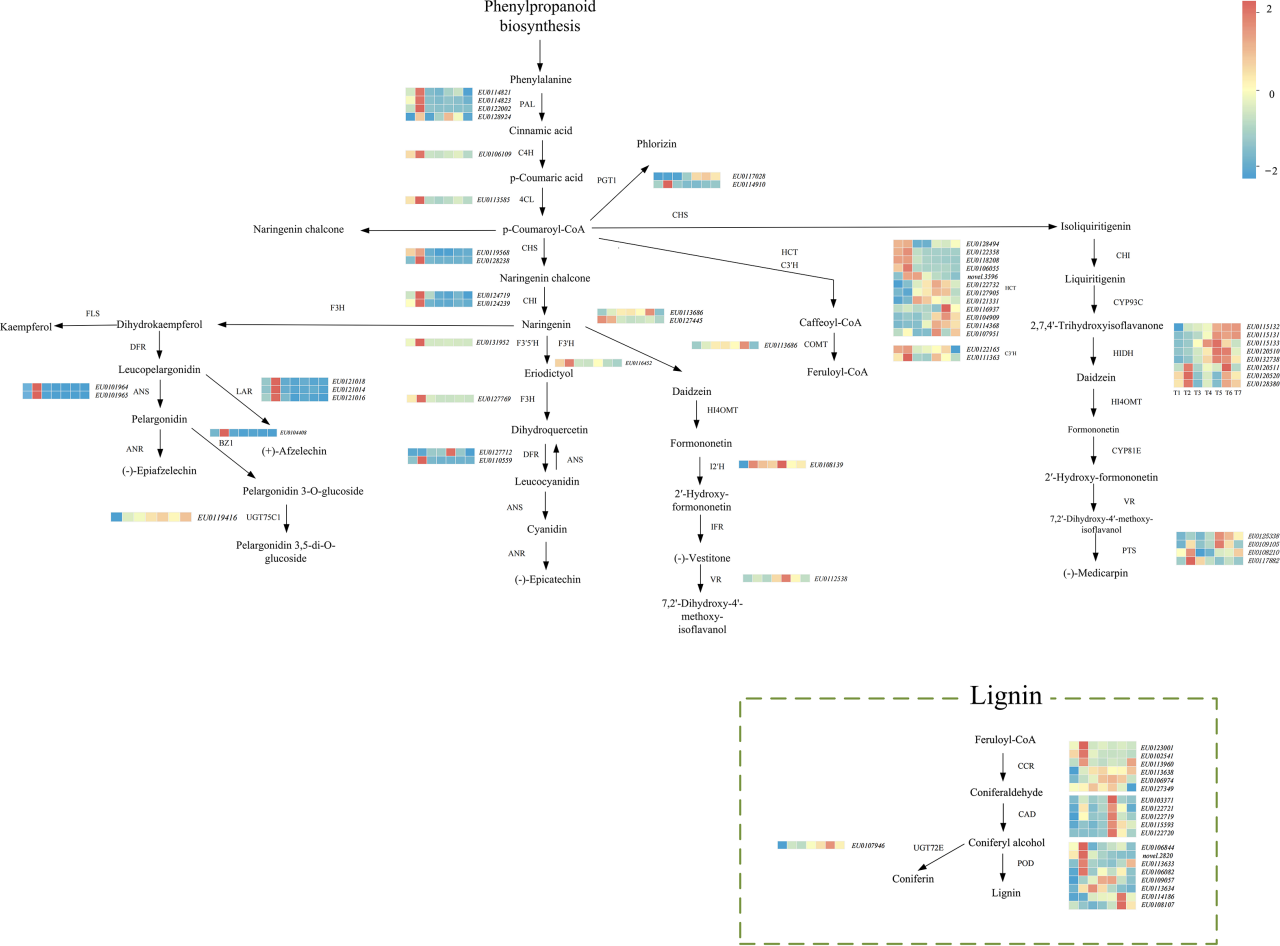
Diagram of the flavonoid biosynthetic pathways in EUL Heatmaps next to each enzyme show expression profiles of the corresponding genes across developmental stages T1-T7. The green dashed box highlights the lignin biosynthetic branch.

K-means clustering showed that the temporal expression patterns of subclasses 1, 2, 5 and 6 were generally consistent with changes in total flavonoid content. These subclasses were enriched in genes involved in flavonoid biosynthesis and spanned key enzymatic steps from backbone formation to subsequent modification reactions. In total, 117 flavonoid-related genes were assigned to these four subclasses. After excluding genes with consistently low expression across all developmental stages (≤10 FPKM at any stage), 75 candidate flavonoid biosynthetic genes were retained. These include genes encoding the enzymes such as PAL, C4H, 4CL, CHS, CHI, F3H, FLS, DFR, ANS, ANR and LAR, as well as genes encoding key enzymes such as UGTs, HCTs and CCRs. Based on previously reported flavonoid biosynthetic pathways, we constructed an integrated pathway map for EUL that combines heatmaps of structural gene expression with the contents of major flavonoid compounds (**Figure 7**). The green dashed box marks the lignin branch, illustrating the diversion of phenylpropanoid intermediates into cell wall–related metabolism. Together, these genes are likely to contribute to the biosynthesis of distinct flavonoid compounds at different developmental stages.

#### 3.2.5 WGCNA-based identification of key genes with flavonoid content

**Figure 8:**
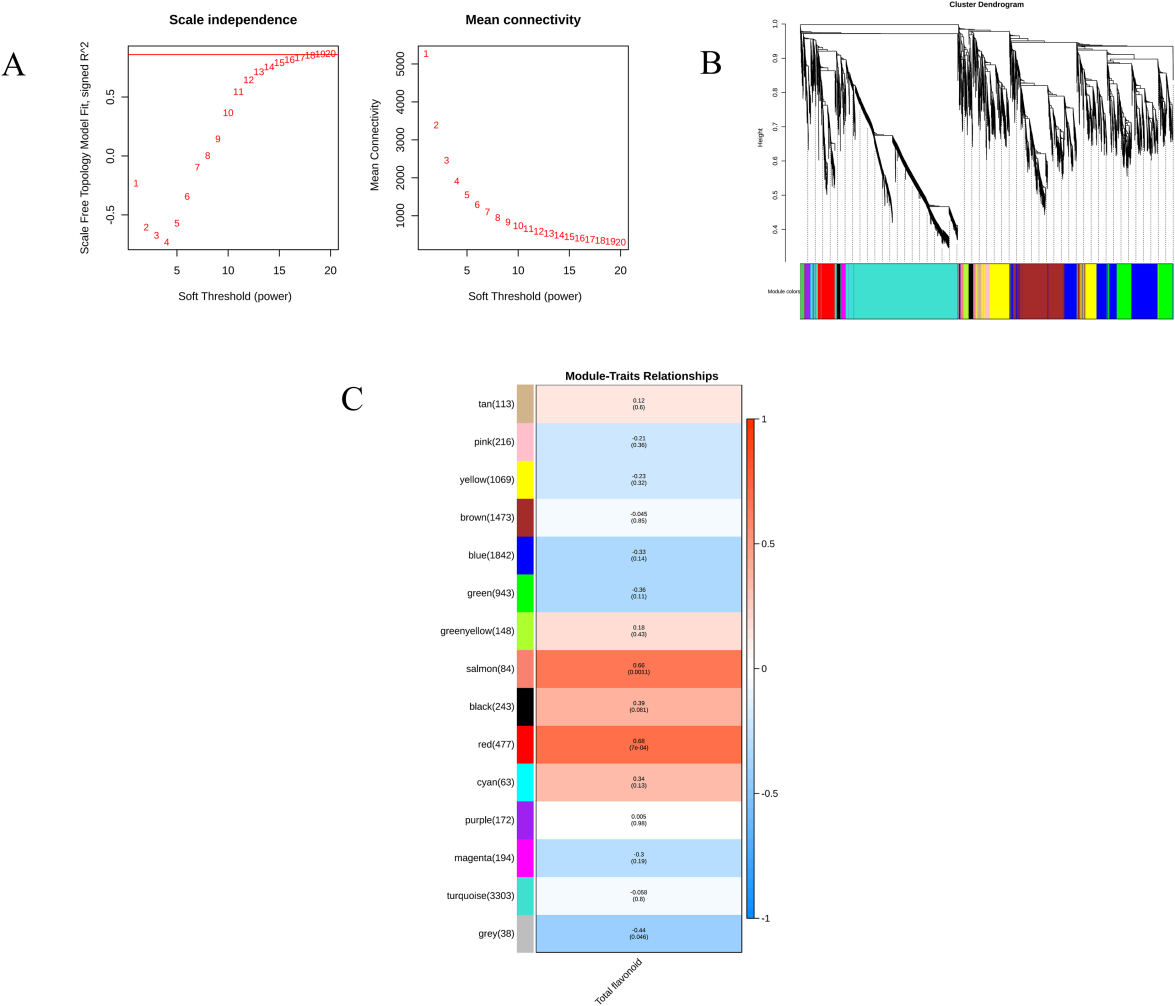
WGCNA analysis linking transcriptome modulesto total flavonoid content in EUL. **(A)** Scale-free topology model fit index (left) and mean connectivity (right) for various soft-thresholding powers. **(B)** Cluster dendrogram of genes, with modules indicated by different colors. **(C)** Module–trait relationships between module eigengenes and total flavonoid content across developmental stages.

We constructed a weighted gene coexpression network (WGCNA) using total flavonoid content and transcriptome data from different developmental stages to identify key modules associated with flavonoid metabolism. With a soft threshold power of β = 18 was chosen (scale-free topology fit index R² ≈ 0.85; **Figure 8A**). 18 modules were obtained (**Figures 8A-C**). Dynamic tree cutting applied to the gene dendrogram initially identified 18 modules, which were further merged at a cut height of 0.25, yielding 14 distinct coexpression modules. Genes that could not be confidently assigned to any module were grouped into the grey module (**Figures 8B, C**). Among these modules, the salmon and red modules showed the positive correlations with total flavonoid content. The genes in these modules were functional associated enriched in the phenylpropanoid and flavonoid metabolism pathways (ko00940–ko00944), and included key structural genes such as *PAL, C4H, CCR, HID* and *CHI* which suggests that total flavonoid accumulation depends on the coordinated action of multiple functional modules. However, the salmon module contained only one gene annotated to phenylpropanoid and flavonoid pathways, so subsequent analyses focused on the red module. Within the red module, 477 genes were detected. Applying thresholds of MM ≥ 0.6 and GS ≥ 0.6 identified 192 genes with both high module membership and a strong association with total flavonoid content (**Supplementary Figure S2**). By further requiring the p-values for both MM and GS to be ≤ 0.05, 183 high-confidence flavonoid-related genes were retained for subsequent functional annotation and KEGG pathway analyses (**Supplementary Table S2**). Among them, 14 genes were annotated to phenylpropanoid and flavonoid biosynthesis pathways (ko00940-ko00944), representing putative structural genes involved in flavonoid metabolism. When an additional cutoff of |MM| ≥ 0.6 (p ≤ 0.05) together with high GS was applied within this subset, two genes remained, *EU0113959* (*CCR1D*, cinnamoyl-CoA reductase) and *EU0125338* (*PTS1*, pterocarpan synthase 1), which showed both high gene significance and high module membership, and were therefore defined as core key genes.

##### 3.2.5.1 Correlation between module eigengenes and flavonoid content

**Figure 9:**
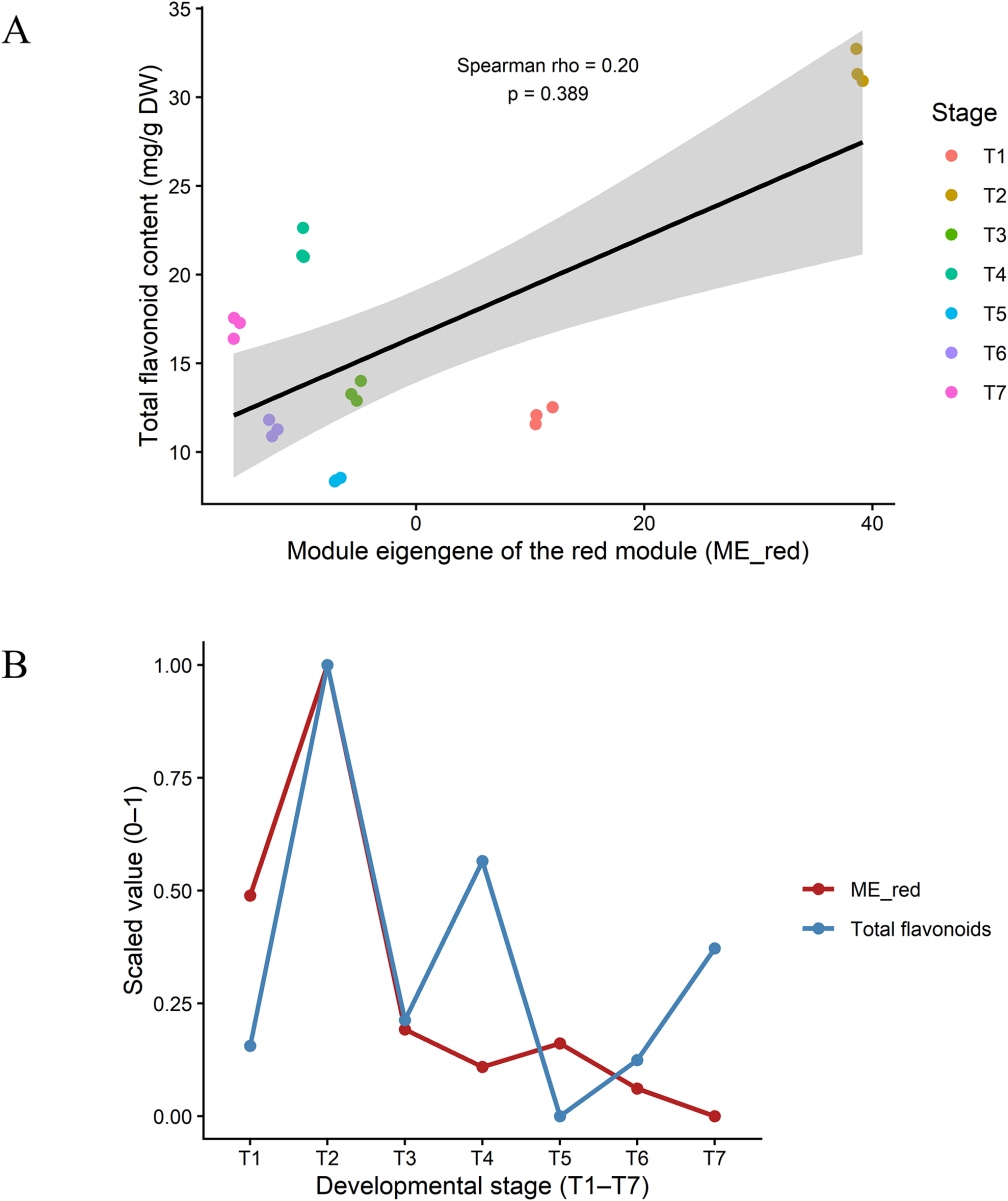
Correlation analysis between the eigengenes of the red module and total flavonoid content. **(A)** Scatterplots of the eigengene of the red module (ME_red) versus total flavonoid content across all samples. Each point represents one biological replicate, colour-coded by developmental stage (T1-T7); **(B)** Scaled temporal profiles of ME_red and total flavonoid content from T1 to T7, showing that ME_red captures the overall trend but not the full magnitude of metabolite fluctuations.

The ability of the red module to explain the spatiotemporal variation in total flavonoid content was further evaluated by deriving a leave-one-out cross-validated (LOOCV) module eigengene (ME_red) from genes annotated to flavonoid-related pathways (ko00940-ko00944) and correlating it with total flavonoid content across all samples. ME_red showed only a weak positive association with total flavonoid content (Spearman’s ρ = 0.20, p = 0.389; **Figure 9A**), indicating that the eigengene of this core flavonoid module explains only a small fraction of the variation in total flavonoid levels.

After scaling both ME_red and total flavonoid content to the range 0–1, their temporal profiles showed a partially similar pattern from T1 to T7, with a transient increase between T1 and T2 followed by a gradual decline (**Figure 9B**). However, the amplitude of changes in ME_red was much smaller than that of total flavonoid content. This temporal mismatch suggests that transcriptional changes in the red module can capture the overall trend of flavonoid accumulation but are insufficient to fully account for the large and rapid fluctuations in metabolite levels. Additional regulatory layers downstream of transcription are therefore likely to play important roles in shaping the dynamic accumulation of flavonoids in EUL.

#### 3.2.6 qRT-PCR validation

**Figure 10:**
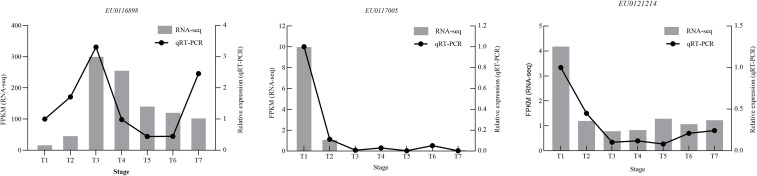
The qRT-PCR analysis across seven developmental stages of EUL.

The reliability of the transcriptome data was assessed by qRT-PCR validation of three flavonoid-related differentially expressed genes *EU0116898, EU0121214* and *EU*0*117005* (**Figure 10**). The qRT-PCR profiles closely the mirrored developmental patterns obtained from the RNA-seq data, although absolute transcript levels at individual stages were not identical. This concordance indicates that the sequencing data are robust.

**Table 1:** The primers for real-time PCR.

Gene name	Forward primer sequence	Reverse primer sequence
GAPDH	CCGAAGATGATTTGGTGTCCACAG	TTCATTGTCGTACCAGGAGACGAG
*EU0116898*	ACATCTGCCGGGAAGACAAA	TCCATGCCCAATTGCCTGAA
*EU0121214*	ACCTCCATTTGCTGTGCTGT	GCATTCACTTTGACCGCCAG
*EU0117005*	CAAAGCCTCACCCCACGTAT	GACGTTGTGCTCTCCTCGAA

**Table 2:** Quality assessment table of RNA-seq data.

Sample	e	GC content (%)	Q30 (%)	Total mapped (%)
T1-1	41987872	48.73	94.62	94.42
T1-2	42798580	48.9	94	94.28
T1-3	43553958	48.35	94.43	94.37
T2-1	40681640	49.15	94.15	93.61
T2-2	46273086	48.48	94	94.07
T2-3	43726102	48.62	94.22	93.98
T3-1	41030568	48.03	94.46	94.42
T3-2	40668794	47.93	94.29	94.32
T3-3	39413302	47.96	94.73	94.36
T4-1	43953236	47.28	94.12	93.98
T4-2	43541378	47.35	94.25	94.14
T4-3	43787846	47.22	94.21	93.95
T5-1	41241762	47.32	94.59	94.27
T5-2	43933490	47.1	94.16	94.08
T5-3	44027292	47.09	94.52	94.19
T6-1	44008970	47.27	94.1	93.91
T6-2	43009620	47.16	94.07	93.80
T6-3	43631116	47.24	94.22	93.79
T7-1	40371190	47.15	94.52	92.77
T7-2	44155246	47.13	94.3	92.55
T7-3	42905658	47.31	94.65	92.10

## 4 Discussion

### 4.1 Seasonal variation of total flavonoids

The temporal dynamics of bioactive compounds in plants are usually shaped by the interplay between developmental processes and seasonal environmental conditions, which is a common feature of secondary metabolism in perennial woody species. By continuously measuring the total flavonoid content of EUL across the full developmental series, we observed a clear seasonal pattern within a single growing season, total flavonoid content reached a primary peak during the rapid leaf expansion stage (T2) in spring, showed a secondary peak in midsummer (T4), and then exhibited a modest increase again in autumn (T7).

The three-year continuous sampling study conducted in Yangling also reported similar seasonal dynamics, with total flavonoid content in EUL peaking in May, showing a secondary maximum in July, and then gradually declining, followed by a slight increase before winter. Even though absolute concentrations were different between the Yangling production regions and our site, the seasonal curve looked much the same in terms of when it rose and fell (**Figure 2**), suggesting that this pattern travels well across years and locations. Temperature and accumulated heat units also differed between Inner Mongolia and Yangling, and they lined up with the between-site shifts in total flavonoids. Taken together, this points to thermal conditions as a plausible factor shaping how much flavonoid accumulates and possibly when it peaks in EUL (Zhang et al., 2013).

As research on flavonoids has progressed, they have been recognized as performing multiple functions in plants, including UV shielding, defence against insects and pathogens, enhancement of cold tolerance, regulation of cell division and participation in broader secondary metabolic networks (Ramaroson et al., 2022; Daryanavard et al., 2023; Tariq et al., 2023; Li et al., 2025). These diverse roles imply that patterns of flavonoid accumulation are closely coupled to leaf developmental stage and seasonal environmental conditions. Total flavonoid content in EUL peaked during the rapid expansion phase from April to May. At this stage, leaves are young and metabolically active, and higher flavonoid levels likely provide extra antioxidant and defence capacity. A comparable pattern has been reported in *Carica papaya* L. where young tissues contain significantly more flavonoids than mature leaves (Misnan et al., 2024). Seasonal and stage-dependent variation is also evident in the woody species *Cyclocarya paliurus* (Chen et al., 2025).

Taken together with findings from other plant species, the second peak observed in July likely reflects an adaptive protective response of EUL to intense summer light and high temperatures, while simultaneously promoting the accumulation of pharmacologically relevant secondary metabolites (Darwish et al., 2021; Ma et al., 2024). The slight increase observed in October, in turn, may be related to pre-winter redistribution and storage of nutrients and antioxidants, helping the plant to better withstand oxidative stress and environmental fluctuations during overwintering (Valares Masa et al., 2016; Mattila et al., 2018).

### 4.2 Functional insights from GO and KEGG enrichment

GO enrichment analysis revealed that different developmental stages of EUL leaves are dominated by processes typical of actively growing tissues. In the BP category, cellular process and metabolic process were consistently over-represented, supporting the view that leaf development is driven by coordinated cell division, expansion and intense primary and secondary metabolism (Sablowski and Carnier Dornelas, 2014; Thomas, 2017). Enriched CC terms related to membranes and organelles suggest that dynamic changes in intracellular compartmentation and membrane transport underpin these developmental transitions, in line with reports emphasizing the importance of organelle function and transport systems in leaf growth and stress acclimation (Zhou et al., 2022; Kalemba et al., 2024; Bauer and Koppers, 2025).

In the MF category, binding functions were most strongly enriched in the majority of pairwise comparisons. This pattern is consistent with the idea that DNA-, RNA- and protein-binding activities, including those of transcription factors, form a core regulatory layer controlling leaf transcriptional programs (Gaudet et al., 2021). Around the T3–T4 transition, however, catalytic activity terms became more prevalent than binding terms. This shift points to a developmental phase in which regulation by enzymes, rather than transcription factors alone—plays an increasingly important role, implying a stronger contribution of metabolic reactions to shaping the phenotype at this stage (Bian et al., 2022).

KEGG pathway enrichment further refined these observations by highlighting distinct functional phases along the developmental series. Early comparisons (e.g. T1 vs T2 and T2 vs T3) showed significant enrichment of ribosome, translation and energy-related pathways, reflecting high demands for protein synthesis and energy supply during rapid tissue expansion. Similar prominence of ribosome-associated pathways has been reported in other species during vigorous growth or under stress, underscoring translational capacity as a key determinant of developmental and environmental responses (Hu et al., 2022; Wang, M. et al., 2025).

As leaves entered the mid-developmental stages, enrichment patterns shifted towards secondary metabolism. Pathways such as flavonoid biosynthesis, flavone and flavonol biosynthesis and anthocyanin biosynthesis were strongly over-represented, indicating that the transcriptional machinery increasingly invests in the production of flavonoids and related phenylpropanoids. Flavonoids contribute to UV screening, antioxidant defence and biotic stress resistance, and also possess diverse pharmacological activities in humans (Liu et al., 2021; Naik et al., 2022; Gao et al., 2023). Activation of these pathways around the T2–T4 stages therefore likely supports both structural and physiological maturation of the leaves and establishes the molecular basis for subsequent accumulation of medicinally relevant flavonoids in EUL.

At later stages, the enrichment profile became dominated by defence- and signaling-related pathways. MAPK signaling, plant hormone signal transduction, circadian rhythm, α-linolenic acid metabolism and benzoxazinoid biosynthesis were all significantly enriched, pointing to a transition from growth-oriented metabolism to enhanced environmental adaptation and defence. α-Linolenic acid metabolism is closely linked to jasmonate-related signaling and defence responses (Yu et al., 2021; Nie et al., 2024), while benzoxazinoids are well-known defence metabolites in cereals and other species (Duan et al., 2022). The concurrent enrichment of MAPK and hormone signaling pathways suggests that EUL leaves gradually assemble a complex regulatory network that integrates developmental cues with external stimuli, thereby strengthening stress resilience towards the later stages of the season (Manna et al., 2023; Zhu et al., 2024; Yao et al., 2024).

Comparison of these transcriptional patterns with the metabolic data indicates that gene expression and metabolite accumulation are not always synchronized. For example, strong enrichment of flavonoid-related pathways does not immediately translate into proportional increases in flavonoid content, and some stages with declining metabolite levels still display pronounced pathway enrichment. Similar temporal mismatches between pathway activation and final flavonoid levels have been documented in other species, where post-transcriptional regulation, enzyme activity, substrate competition, transport and metabolite turnover all influence the final steady-state concentration of flavonoids (Guo et al., 2019; Tao et al., 2017; Wang et al., 2024). These observations are consistent with the WGCNA-based analysis, in which the red module eigengene (ME_red) showed only a weak correlation with total flavonoid content despite capturing the overall developmental trend. Taken together, the GO and KEGG results indicate that flavonoid accumulation in EUL is shaped by a multi-layered regulatory system in which transcriptional control is necessary but not sufficient, and must be complemented by downstream biochemical and physiological regulation.

### 4.3 K-means clustering of flavonoid pathway genes

The K-means clustering of flavonoid pathway genes identified a cluster enriched for structural enzymes whose expression profiles closely paralleled the dynamic changes in total flavonoid content. This cluster includes upstream genes such as *PAL* and *CCR*, the core backbone enzymes *CHS*, *CHI* and *F3H*, and downstream enzymes *DFR*, *ANS*, *ANR* and *LAR*, together spanning almost all key structural steps of the flavonoid pathway. In addition, the same cluster contains 2-hydroxyisoflavanone dehydratase (*HID*, K13258), isoflavone 2′-hydroxylase (K13260), vestitone reductase (K13265) and pterocarpan synthase (*PTS1*, K25075), which are associated with isoflavonoid and pterocarpan branches, indicating that EUL harbors a relatively complete metabolic network for flavonoids and their derivatives. Consistent with our findings, an integrated transcriptome–metabolome analysis in *Codonopsis* showed that structural genes such as *ANS*, *CHI*, *CHS* and *F3H* occupy central positions in the regulation of flavonoid biosynthesis (Liu et al., 2025). Together, these results suggest that a pathway backbone extending from *PAL* at the pathway entry step, through *CHS*, *CHI* and *F3H*, to the downstream reductases and synthases *DFR*, *ANS*, *ANR* and *LAR*, with extensions into isoflavonoid and pterocarpan branches, represents a conserved core module across species and supports the reliability of our annotation and functional inferences for key flavonoid biosynthetic genes in EUL.

### 4.4 WGCNA reveals hub genes and temporal mismatch between transcription and flavonoid accumulation

The WGCNA analyses showed that in the red module, which was significantly correlated with total flavonoid content, flavonoid accumulation (ko00940-ko00944) in EUL leaves is not dominated by any single structural gene, but is instead coordinated by a set of genes with complementary functions. Within this module, combining gene significance, module membership and KEGG annotation with respect to total flavonoids, we identified several key regulatory nodes, among which EU0113959 (CCR1D, cinnamoyl-CoA reductase) and EU0125338 (PTS1, pterocarpan synthase 1) stand out as pivotal genes at metabolic branch points. CCR1D is positioned at an early divergence point in the phenylpropanoid pathway, directing activated cinnamoyl-CoA esters towards lignin and related phenolic derivatives; changes in its activity are therefore expected to influence the size and allocation of the shared phenylpropanoid precursor pool, indirectly constraining the carbon flux available for flavonoid biosynthesis. PTS1 catalyzes the formation of lupin-type pterocarpans in the isoflavonoid branch, suggesting that part of the phenylpropanoid flux in EUL is diverted into specialized defence-related derivatives rather than being channeled solely into classical flavonoid end-products such as flavonols and anthocyanins. It should be emphasized that, although CCR1D and PTS1 act as “valves” for carbon-flow partitioning, the time-series variation in total flavonoid content is only weakly and positively correlated with the red module eigengene (ME_red; ρ= 0.20), and the amplitudes of change differ markedly. This indicates that, while synergistic transcriptional regulation within the red module helps to define the overall developmental trend of flavonoid accumulation, it cannot by itself account for the pronounced seasonal fluctuations in total flavonoid content, revealing a clear temporal mismatch between gene expression and metabolite levels. Such temporal decoupling between transcription and metabolite accumulation has been reported in several plant species. In Ginkgo biloba leaves (Temporospatial Flavonoids Metabolism Variation in Ginkgo biloba Leaves), transcriptional activation of flavonoid biosynthesis genes tended to precede substantial changes in flavonoid and flavonoid-derivative contents, with some metabolite peaks even occurring after structural gene expression had declined back towards baseline. In Tartary buckwheat seeds (An Evaluation of the Absolute Content of Flavonoids and the Identification of Their Relationship with the Flavonoid Biosynthesis Genes in Tartary Buckwheat Seeds), different flavonoid fractions displayed markedly distinct developmental accumulation trajectories, with some showing strong positive correlations with key structural genes, whereas others showed negative or delayed responses, reflecting stage-specific dynamic regulation. Viewed alongside these studies, the pattern observed in EUL, relatively modest transcriptional fluctuations in the red module but marked seasonal changes in total flavonoid content supports the notion that flavonoid accumulation is governed by a multi-layered regulatory network. In this framework, transcriptional regulation primarily sets the developmental trajectory, while enzyme activity, substrate partitioning, transmembrane transport and metabolite turnover together shape the final flavonoid levels and their seasonal variation.

We experimentally evaluated two commonly used reference genes, GAPDH and ACTIN, and found that GAPDH exhibited higher stability across the seven developmental stages at both the transcriptome (FPKM) and qPCR (Ct values) levels. In addition, RT-qPCR expression patterns normalized to GAPDH were more consistent with the RNA-seq expression profiles of the target genes than those normalized to ACTIN, suggesting that GAPDH is a suitable reference gene under the experimental conditions of this study. These observations support the use of GAPDH as a single, experimentally validated reference gene in this work.

## 5 Conclusion

We quantified total flavonoid content in EUL across seven developmental stages and integrated these data with transcriptome profiles to characterize the developmental dynamics of flavonoid accumulation. In total, 14,827 differentially expressed genes were identified, of which 9,816 were associated with specific stages (T1-T7). By combining KEGG annotation with K-means clustering, we delineated a cluster of flavonoid pathway genes, indicating that EUL harbors a relatively complete metabolic network for flavonoids and their derivatives. WGCNA further identified a red coexpression module that was significantly correlated with total flavonoid content; within this module, *CCR1D (EU0113959)* and *PTS1 (EU0125338)* emerged as key genes at key metabolic branch points, reflecting fine partitioning of phenylpropanoid flux among lignin, core flavonoids and specialized isoflavonoid derivatives. However, the module eigengene showed only a weak and temporally offset correlation with total flavonoid content, suggesting that transcriptional regulation, although crucial for establishing the developmental trend of flavonoid accumulation, is not sufficient by itself to explain the pronounced seasonal fluctuations. Overall, these results support a multilayer regulatory model in which flavonoid metabolism in EUL is shaped jointly by transcriptional control, enzyme activity, substrate allocation, transmembrane transport and metabolite turnover. This study provides an integrated framework for understanding flavonoid biosynthesis and its regulatory network in EUL, and offers a theoretical basis for the precise manipulation of flavonoids and other key metabolites, as well as for optimizing harvest timing.

## Data Availability

The authors acknowledge that the data presented in this study must be deposited and made publicly available in an acceptable repository, prior to publication. Frontiers cannot accept an article that does not adhere to our open data policies.
