# Supplementary material for: Transcriptome mining of key genes involved in seasonal changes of total flavonoids in Eucommia ulmoides leaves
Source: Front Plant Sci. 2026 Feb 17;17:1718338. doi: 10.3389/fpls.2026.1718338 (PMC12954871; doi:10.3389/fpls.2026.1718338)
Supplement: Supplementary file 1 [file DataSheet1.docx]

Table S1 Statistics of functional annotation results of DEGs

| DEGs Set | UP | Down | Total |
| --- | --- | --- | --- |
| T1 vs T2 | 2391 | 2964 | 5355 |
| T2 vs T3 | 1415 | 2343 | 3758 |
| T3 vs T4 | 485 | 406 | 891 |
| T4 vs T5 | 1027 | 400 | 1427 |
| T5 vs T6 | 448 | 604 | 1052 |
| T6 vs T7 | 1005 | 1638 | 2643 |


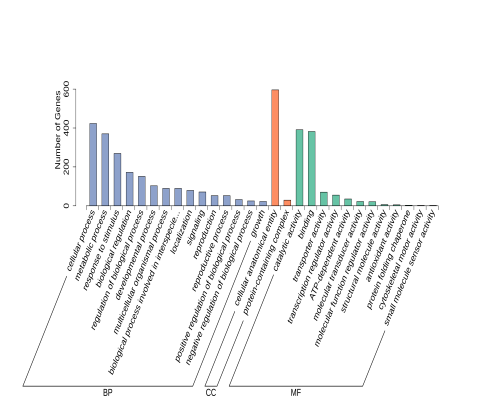

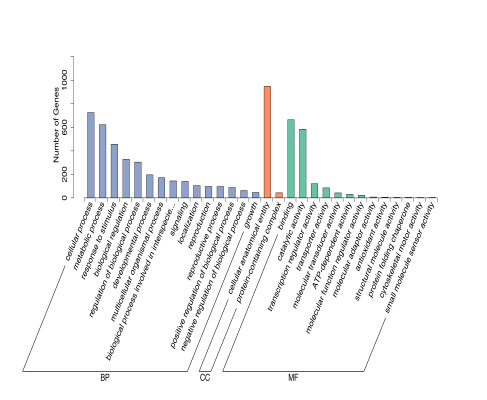

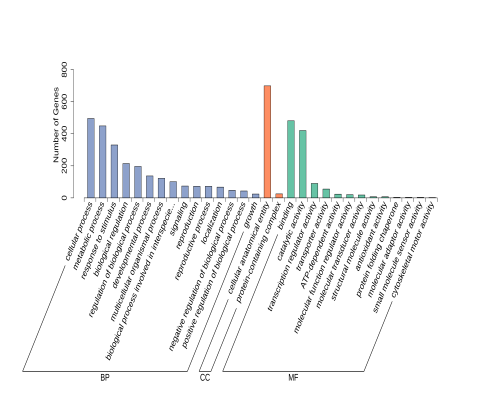

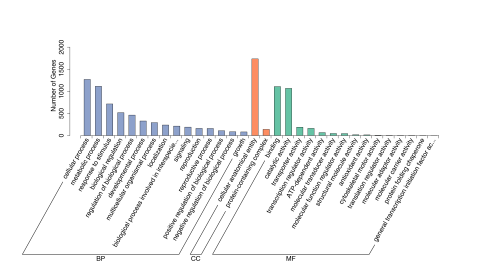

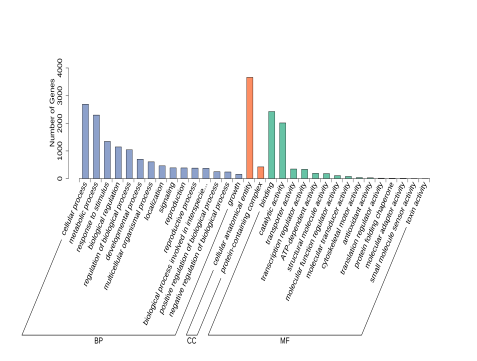


T1 vs T2

Fig.3-1 GO annotation classification statistical map of DEGs


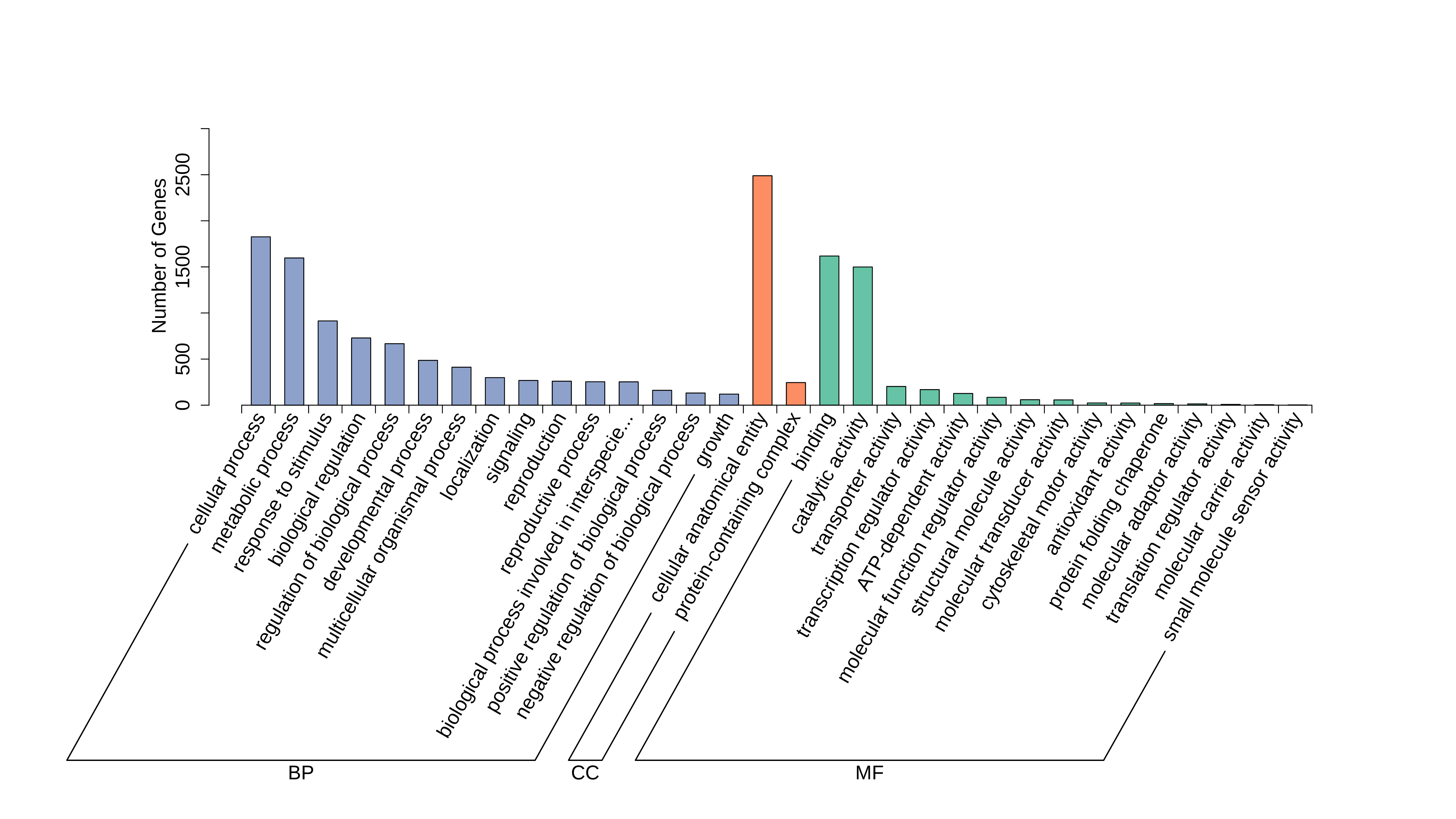


Figure S1. GO functional classification of annotated genesat different developmental stages

T2 vs T3

Fig.3-1 GO annotation classification statistical map of DEGs

T3 vs T4

Fig.3-1 GO annotation classification statistical map of DEGs

T4 vs T5

Fig.3-1 GO annotation classification statistical map of DEGs

T5 vs T6

Fig.3-1 GO annotation classification statistical map of DEGs

T6 vs T7

Fig.3-1 GO annotation classification statistical map of DEGs

A

B

C

D

E

F


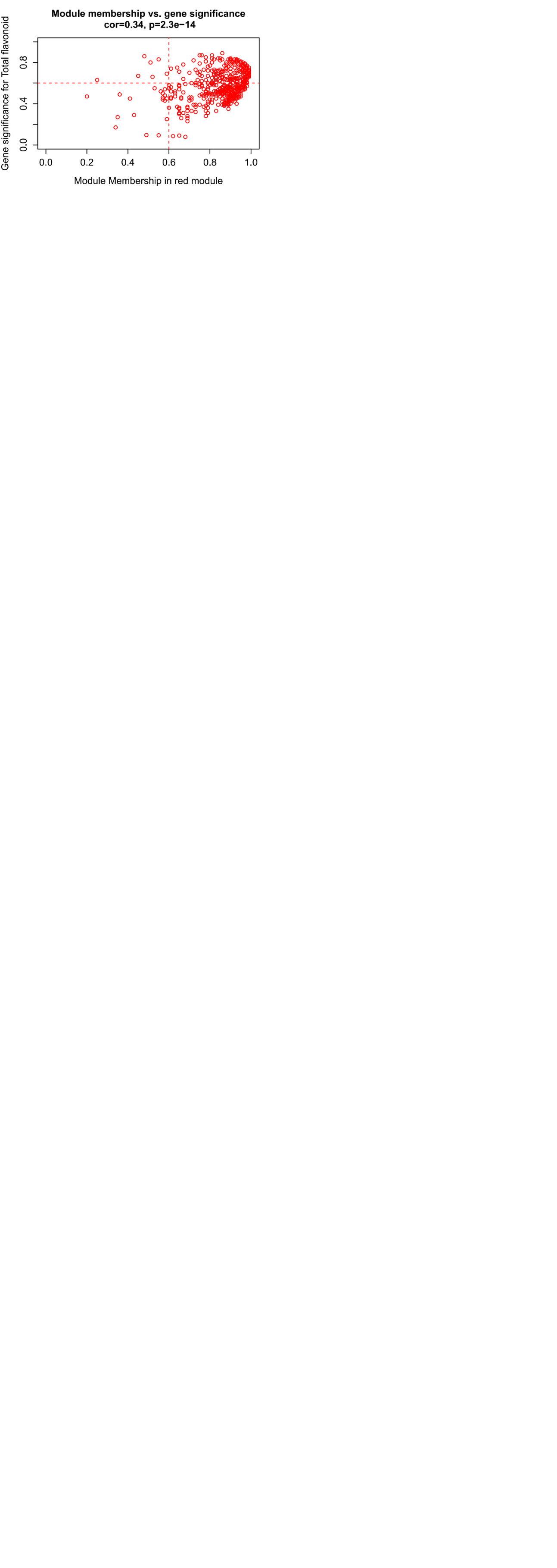


Figure S2. In the red module, 477 genes were identified in total. Using MM ≥ 0.6 and GS ≥ 0.6 as thresholds, 192 genes with both high module membership and gene significance for total flavonoid content were obtained
